# Negative Inotropic Effect of BGP-15 on the Human Right Atrial Myocardium

**DOI:** 10.3390/jcm9051434

**Published:** 2020-05-12

**Authors:** Nóra Lampé, Dániel Priksz, Tamás Erdei, Mariann Bombicz, Rita Kiss, Balázs Varga, Judit Zsuga, Tamás Szerafin, Zoltán Csanádi, György Balla, József Balla, Zoltán Szilvássy, Rudolf Gesztelyi, Béla Juhász

**Affiliations:** 1 Department of Pharmacology and Pharmacotherapy, Faculty of Medicine, University of Debrecen, H-4032 Debrecen, Hungary; lampenori@gmail.com (N.L.); priksz.daniel@pharm.unideb.hu (D.P.); erdei.tamas@pharm.unideb.hu (T.E.); bombicz.mariann@pharm.unideb.hu (M.B.); kiss.rita@med.unideb.hu (R.K.); varga.balazs@pharm.unideb.hu (B.V.); szilvassy.zoltan@med.unideb.hu (Z.S.); gesztelyi.rudolf@pharm.unideb.hu (R.G.); 2 Department of Health Systems Management and Quality Management for Health Care, Faculty of Public Health, University of Debrecen, H-4032 Debrecen, Hungary; zsuga.judit@med.unideb.hu; 3 Department of Cardiology and Cardiac Surgery, Clinical Center, University of Debrecen, H-4032 Debrecen, Hungary; szerafin@med.unideb.hu (T.S.); csanadi.zoltan@med.unideb.hu (Z.C.); 4 Department of Pediatrics, Clinical Center, University of Debrecen, H-4032 Debrecen, Hungary; balla@med.unideb.hu; 5 Department of Internal Medicine, University of Debrecen, Debrecen, H-4032 Debrecen, Hungary; balla@belklinika.com

**Keywords:** BGP-15, propranolol, atrium, heart, inotropic effect, patients

## Abstract

Cardiovascular morbidity and mortality carry great socioeconomic burden worldwide that mandates the development of new, efficacious therapeutic agents with limited adverse effects. O-(3-piperidino-2-hydroxy-1-propyl) nicotinic acid amidoxime (BGP-15) is a known, well-tolerable drug candidate that exerts beneficial effects in several disease models. As BGP-15 has a significant structural similarity with propranolol, it arose that BGP-15 might also have a direct effect on the heart. Thus, in the present work, we investigated the effect of BGP-15 and propranolol on the contractility of isolated, paced, human right atrial samples (obtained from patients undergone open-heart surgery), with or without previous isoproterenol (ISO) stimulation (evoking an indirect or direct effect, respectively). We found that both BGP-15 and propranolol exerted direct as well as indirect negative inotropic effects on the atrial myocardium, reaching similar maximal response. However, BGP-15 had considerably smaller potency than propranolol regarding both types of negative inotropy. In addition, BGP-15, in contrast to propranolol, had a significantly greater indirect negative inotropic effect on samples exhibiting strong response to ISO. Moreover, the indirect negative inotropic effect of BGP-15 was significantly greater on samples derived from diabetic patients than on samples obtained from non-diabetic ones. Our results suggest that the enhanced ISO sensitivity is associated with the diabetic state, and BGP-15 exerts greater negative inotropic effect on the human atrial myocardium in both conditions (as compared to the atrial tissue that is not ISO oversensitive and/or diabetic). Additionally, the negative inotropic effects of BGP-15 and propranolol seem to be mediated by in part different molecular pathways in the atrial myocardium.

## 1. Introduction

The prevalence of cardiovascular diseases (CVDs)—including high blood pressure, atrial fibrillation (AF), and heart failure—is continuously increasing worldwide, predominantly in the developed countries. Improper (e.g., sedentary) lifestyle, stress, and obesity all contribute to the likelihood of developing CVDs [1]. In 2014, every third person older than 20 years suffered from high blood pressure in the USA. In 10 years (until 2015), the mortality rate attributable to high blood pressure increased by 10.5%, showing a deteriorating trend [1]. Accordingly, beyond prevention, the therapy of CVDs must also be addressed. Most of the widely available therapeutic options have limited efficiency or are accompanied by several undesirable effects [2,3,4].

BGP-15 (O-(3-piperidino-2-hydroxy-1-propyl) nicotinic acid amidoxime), a derivative of propranolol, was originally developed to improve nephro- and neurotoxicity of cisplatin as a chemoprotectant [5,6,7,8]. This protective effect, the mechanism of which remains unknown yet, occurred at 100–200 mg/kg/day oral dose. However, BGP-15 was found to elicit an insulin-sensitizing effect as well, observed at much lower doses (2–3 mg/kg/day). In these human clinical trials, BGP-15 proved to be well-tolerable, without any significant adverse effect [9,10,11,12]. The insulin sensitizer action of BGP-15 was attributed to the expression of heat shock protein 70 (HSP70) and 72 (HSP72) [13,14,15,16,17,18], although some authors debated this mechanism [19]. Beyond this, Sapra et al. found that, in heart failure and atrial fibrillation, BGP-15 reduced episodes of arrhythmia, attenuated atrial enlargement, and improved cardiac function by inducing phosphorylation of the insulin-like growth factor receptors [19]. The protective effect of BGP-15 against ischemia-reperfusion injuries was also noticed [20,21]. Nevertheless, the mechanism of action for BGP-15 is not yet clarified entirely.

Propranolol, beyond its well-known cardiac effects as a beta-blocker, was shown to affect the vascular tension as well. Interestingly, in aorta isolated from diabetic rabbits, propranolol blunted the constriction induced by norepinephrine, while it had no significant effect on the healthy aorta. This action of propranolol, a kind of “antidiabetic” activity, was attributed to its beta-blocker property [7,22].

Because of its structural similarity to propranolol, it cannot be excluded that BGP-15 also exerts an effect on the β-adrenoceptors. Thus, the main aim of the present investigation was to explore the inotropic effect of BGP-15 on the human myocardium, and to compare it with that of propranolol, the parent molecule of BGP-15. For this purpose, samples were collected from the right atrium of patients who had undergone open-heart surgery. Although sampling from the left ventricle would be the optimal choice, due to surgical and ethical reasons, small right atrial trabeculae carneae were obtained.

## 2. Materials and Methods

### 2.1. Patients, Tissue Samples, and Experimental Design

Patients admitted to the Department of Cardiology and Cardiac Surgery (University of Debrecen, Debrecen, Hungary), from 1 October 2016 to 1 February 2018, for an open-heart surgery (mostly to carry out coronary bypass or valve prosthesis implantation), were asked to participate in this investigation. The study was designed in accordance with the principles of the Declaration of Helsinki, and it was approved by the Medical Research Council (7-8 Szechenyi Istvan ter, Budapest, Hungary) with ethical approval reference number ETT TUKEB 39762-3/2016/EKU (19 September 2016). Written informed consent was obtained from each participant prior to inclusion.

**Figure 1:**
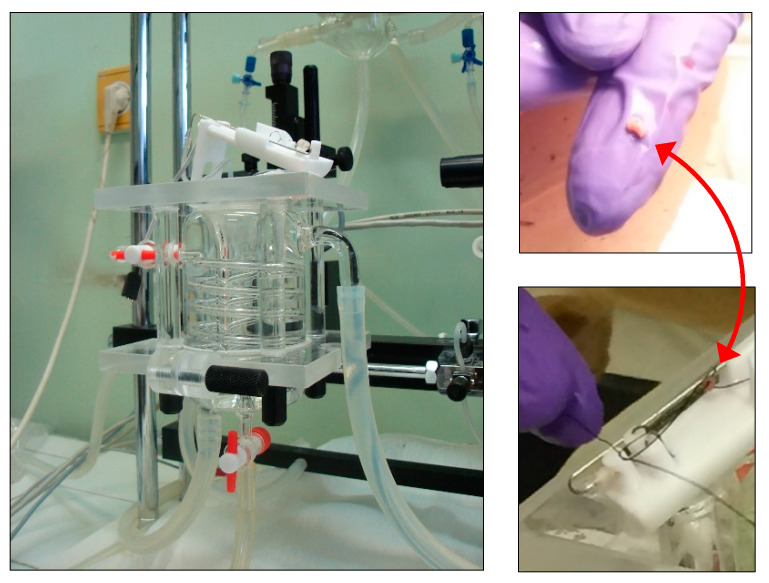
An isolated-organ bath with a (white) tissue holder placed on its top (left panel), and a trabecula carnea, indicated by a double-pointed red arrow, on a finger (right upper panel) and on a tissue holder (right lower panel).

During cannulation of the right atrium (being a part of the routine surgical activity), a small portion of the atrial wall was dissected from the patients under general anesthesia, just before cardioplegia. The atrial sample was placed into ice-cold, oxygenated Krebs solution. From the sample, one trabecula carnea was isolated (diameter: 0.7–1 mm; length: 3–6 mm) and mounted at 10 mN resting tension in a 10 mL vertical organ chamber (Experimetria TSZ-04, Experimetria Kft, Budapest, Hungary) containing Krebs solution, oxygenated with 95% O_2_ and 5% CO_2_ (35.5 °C; pH = 7.4) (Figure 1). The trabecula was paced by a platinum electrode (1 Hz, 2 ms, 7–10 V) by means of a programmable stimulator (Experimetria ST-02, Experimetria Kft, Budapest, Hungary) and power amplifier (Experimetria PST-02, Experimetria Kft, Budapest, Hungary). The contractile force was characterized by the amplitude of the isometric twitches, which were measured by a transducer (Experimetria SD-01, Experimetria Kft, Budapest, Hungary) and strain gauge (Experimetria SG-01D, Experimetria Kft, Budapest, Hungary), and recorded by a polygraph (Medicor R-61 6CH Recorder, Medicor Művek, Budapest, Hungary).

### 2.2. Materials

The following chemicals were used: adenosine, isoproterenol (isoprenaline; ISO), O-(3-piperidino-2-hydroxy-1-propyl) nicotinic acid amidoxime hydrochloride (BGP-15) and propranolol, purchased from Sigma (St. Louis, MO, USA).

All chemicals (excepting ISO) were dissolved in modified Krebs–Henseleit buffer (Krebs solution) containing (in mM): NaCl: 118, KCl: 4.7, CaCl_2_: 2.5, NaH_2_PO_4_: 1, MgCl_2_: 1.2, NaHCO_3_: 24.9, glucose: 11.5, ascorbic acid: 0.1 (dissolved in redistilled water). For dissolving ISO, physiological (0.9 m/v %) NaCl solution was used. Adenosine was dissolved at 36 °C, while other chemicals at room temperature. Stock solutions were diluted with Krebs solution. Stock solutions were freshly prepared daily.

### 2.3. Protocols

The atrial samples were equilibrated in Krebs solution for 45 min, and then a cumulative adenosine concentration-response (E/c) curve was generated followed by a 30-min wash-out period. Next, another cumulative adenosine E/c curve was constructed followed by a 15-min wash-out period. Afterwards, a third cumulative adenosine E/c curve was generated. (Generation of three, rather than one, adenosine E/c curves at the beginning of the *ex vivo* investigation considerably enhanced the viability of the human atrial samples.) After 15-min wash-out, samples were randomized into two main groups: one that received ISO subsequently, and another one that did not (Direct group).

In the Direct group, atria were subjected to a cumulative E/c curve with BGP-15 or propranolol (forming the BGP-15 and the Propranolol subgroup, respectively).

**Figure 2:**
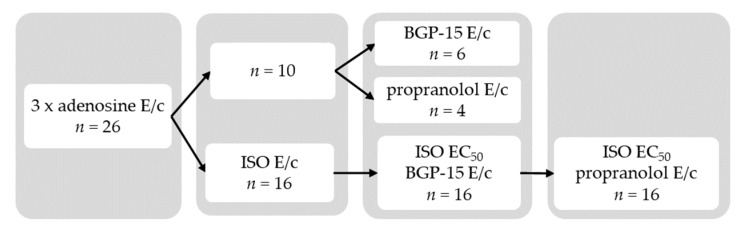
Scheme of protocols of the present study. E/c: concentration-response curve; ISO: isoproterenol; EC_50_: half maximal effective concentration; BGP-15: O-(3-piperidino-2-hydroxy-1-propyl) nicotinic acid amidoxime hydrochloride.

In the other sample set, atria received a cumulative ISO E/c curve (from 1 nmol/L to 100 µmol/L, but the curve construction was immediately stopped if the contractile force showed a sudden decrease in response to an ISO dose, after a previous increase). Following a 30-min wash-out, ISO was administered to reach its half maximal effective concentration (EC_50_) in the bathing medium (regarding the positive inotropic response of the particular trabecula, see below). After stabilization of the contractile force, a cumulative BGP-15 E/c curve was generated. After a 50-min wash-out, samples were stimulated with the EC_50_ of ISO again, and then a cumulative propranolol E/c curve was constructed (Figure 2).

The ISO EC_50_ was individually determined by reading the concentration corresponding to the half of the maximal effect from the ISO E/c curve of each sample. For E/c curves missing their starting (highly unsaturated) part, EC_50_ values were estimated approximatively (for safety, deciding smaller rather than greater value).

### 2.4. Data Analysis

Each atrial sample was required to meet two criteria in order to be included in the further evaluation: (1) The resting contractile force had to reach 1 mN. This criterion had to be obeyed first at the end of the wash-out period after the third adenosine E/c curve, and then at the end of every wash-out period (before the generation of an E/c curve). (2) The mechanical activity of the paced atrial trabecula had to be regular. Additionally, atria showing paradoxical or irregular inotropic response to ISO were also excluded from the evaluation (for more detail, see the Section 3).

Normality of data was checked using Shapiro–Wilk normality test. Two data sets, if passed the normality test, were compared with unpaired *t* test (without or with Welch’s correction, depending on homogeneity or heterogeneity of variances, respectively). For non-Gaussian data, Mann–Whitney U test was used. To compare more than two data sets, one-way ANOVA followed by Tukey post-testing was applied (after verifying the Gaussian data distribution). The linear relationship of two data sets was analyzed with Pearson or Spearman correlation in the case of Gaussian or non-Gaussian distribution, respectively. For the sake of illustration, linear regression was also performed. To assess the relationship between two binomial data sets, Fisher’s test was used. Difference of means (or medians) was considered significant at *p* < 0.05.

Statistical analysis was performed with GraphPad Prism 8.4.2 for Windows (GraphPad Software Inc., La Jolla, CA, USA), while other calculations were made by means of Microsoft Excel 2016 (Microsoft Co., Redmond, WA, USA).

## 3. Results

### 3.1. Contractile Force of the Right Atrial Samples

Most of the right atrial myocardium samples displayed weak or no mechanical activity before the first adenosine E/c curve. In turn, at the end of the wash-out period following the third adenosine E/c curve, the samples reached the maximum of their contractile force. For most samples, the resting contractile force remained the same or at least showed only a moderate decline during the subsequent experiments (regarding the last phase of wash-out periods between two E/c curves).

**Figure 3:**
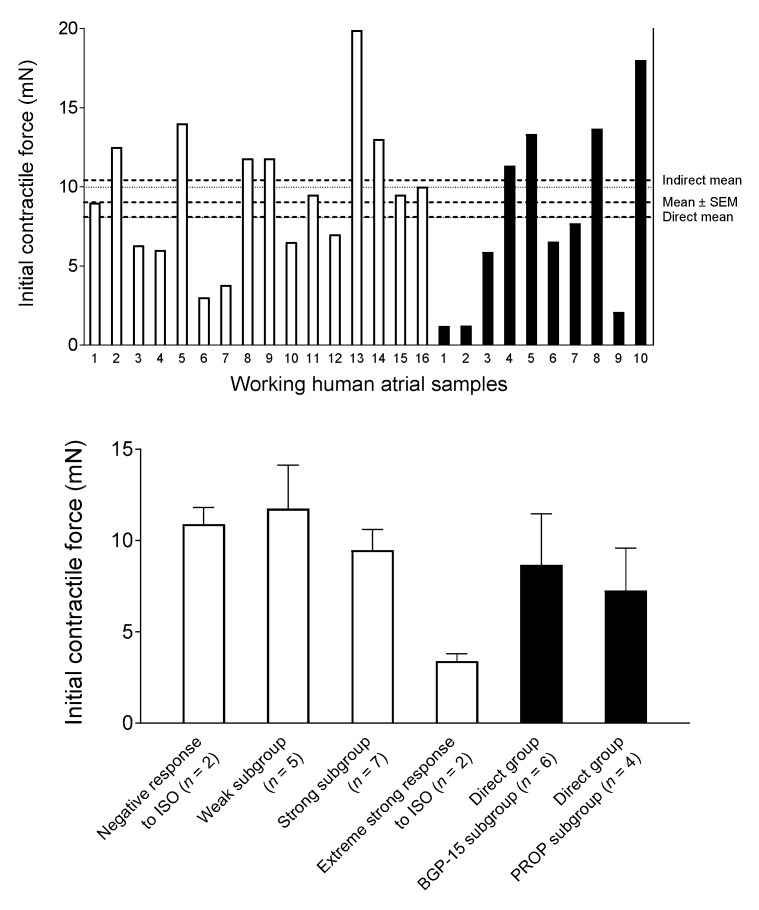
The contractile force of the human right atrial samples at the end of the wash-out period after the third adenosine E/c curve. The upper panel shows the individual values (with the overall mean ± SEM, and with the mean values for the Direct and Indirect groups), while the lower panel denotes the different data sets (mean + SEM). The open columns represent samples that were subjected to isoproterenol (ISO) in a later phase of the experiments. In contrast, the filled columns denote samples without any subsequent ISO treatment. E/c: concentration-response; SEM: standard error of the mean; PROP: propranolol; BGP-15: O-(3-piperidino-2-hydroxy-1-propyl) nicotinic acid amidoxime hydrochloride.

The contractile force, measured at the end of the wash-out period after the third adenosine E/c curve, was used to characterize the naïve contractility of the samples. The averaged contractile force did not differ significantly between atria that were (*n* = 16) and were not (*n* = 10) stimulated with ISO in a later phase of the investigation (Figure 3).

In the case of atria never treated with ISO (Direct group), there was no significant difference between contractility of its two subgroups, the BGP-15 subgroup (*n* = 6) and Propranolol subgroup (*n* = 4).

Within atrial samples subjected to ISO, according to their response to ISO, four types were identified: samples that gave a negative inotropic response (*n* = 2), weak positive inotropic response (*n* = 5), strong positive inotropic response (*n* = 7) and extremely strong positive inotropic response (*n* = 2) (for more detail, see below the Section 3.3). Hereinafter, the atria producing weak positive inotropic response (Weak subgroup) and strong positive inotropic response (Strong subgroup) were pooled as the Indirect group (*n* = 12). (Overall, the Indirect group consisted of atrial samples showing positive inotropic response to ISO that was greater than 0% and was not greater than 100%. The reason for this data pooling is that these two subgroups contain the samples free from any paradoxical or irregular behavior.) The contractile force of samples in the Weak subgroup was greater than that in the Strong subgroup, although this difference did not reach the level of statistical significance. In summary, the naïve contractility of samples in the present study was similar (apart from the two samples giving an extremely strong positive inotropic response to ISO) (Figure 3).

### 3.2. Response to Adenosine

**Figure 4:**
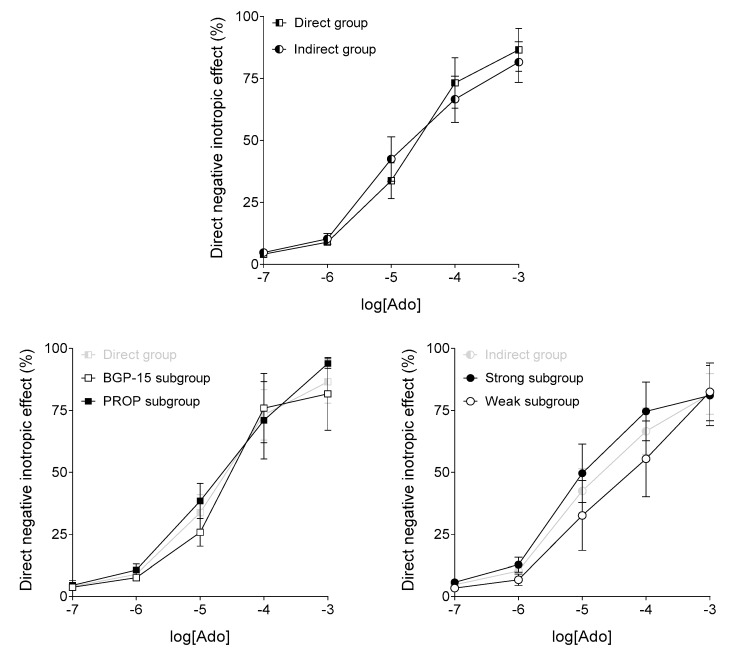
The effect of adenosine (Ado) on the human right atrial samples (based on the third adenosine concentration-response (E/c) curve), not stimulated (Direct group) or stimulated (Indirect group) with isoproterenol (ISO) in a later phase of the experiments. The axis *x* shows the common logarithm of the molar concentration of adenosine, and the axis *y* denotes the effect as a percentage decrease of the initial contractile force. (As any ISO treatment occurred subsequently, herein direct negative inotropic effects are shown in each group.) The symbols represent the effect of adenosine averaged within the groups or subgroups (±SEM). BGP-15, PROP, Weak and Strong in the name of the subgroups refer to events occurred in a later phase of the investigation. SEM: standard error of the mean; BGP-15: O-(3-piperidino-2-hydroxy-1-propyl) nicotinic acid amidoxime hydrochloride; PROP: propranolol.

Responsiveness of the atrial samples to adenosine was evaluated based on the third adenosine E/c curve. Regarding the adenosine-evoked direct negative inotropic effect, there was no significant difference between the Direct and Indirect groups (names of which refer to the absence or presence of subsequent ISO stimulations, respectively). Also, within the two groups, the different subgroups (also distinguished based on subsequent events) did not differ significantly from each other (Figure 4). Thus, the susceptibility of samples to adenosine was similar.

### 3.3. Response to Isoproterenol

ISO elicited a positive inotropic effect from the atrial samples (except for two samples). The positive inotropic response was categorized to be weak, if its maximum did not reach 50% (i.e., an extra 50% in addition to the initial value), strong, if the maximum was between 50–100%, and extremely strong, if the maximum was above 100%. As mentioned above, samples giving negative inotropic and extremely strong positive inotropic responses were excluded from the further analysis.

**Figure 5:**
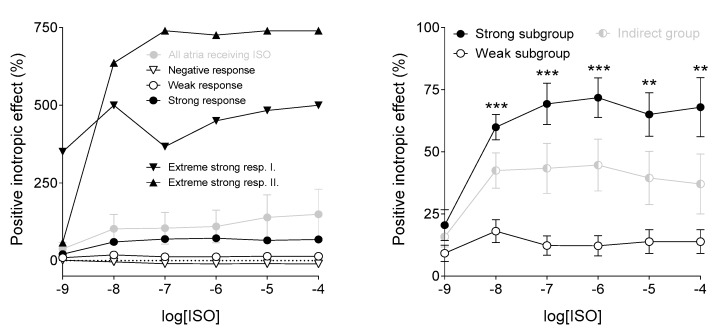
The effect of isoproterenol (ISO) on the human right atrial samples. The axis *x* shows the common logarithm of the molar concentration of ISO, and the axis *y* denotes the effect as a percentage increase of the initial contractile force (in addition to the original level). The symbols represent the effect of ISO averaged within the different data sets (±SEM), and, on the left panel, the individual responses of two samples (Extremely strong resp. I. and Extremely strong resp. II). The right panel shows the Indirect group and its two subgroups. Asterisks indicate the significance level of differences between responses of the two subgroups to the specific ISO concentrations (**: *p* < 0.01; ***: *p* < 0.001). SEM: standard error of the mean.

The positive inotropic effect of ISO was significantly higher in the Strong subgroup than in the Weak subgroup at most ISO concentrations (Figure 5).

**Figure 6:**
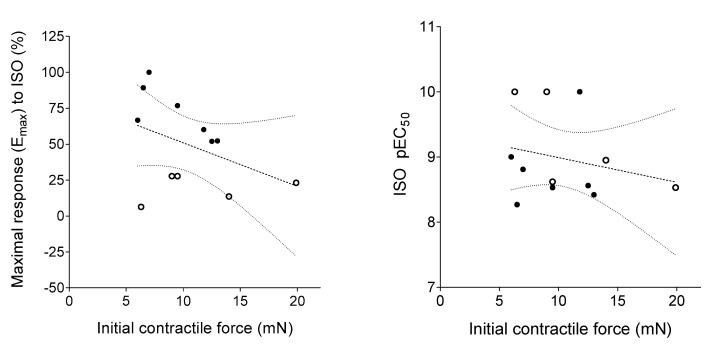
Relationship of the initial contractile force of the human right atrial samples in the Indirect group (measured in millinewton at the end of the wash-out period following the third adenosine E/c curve) with the maximal positive inotropic effect of isoproterenol (ISO) (left panel), furthermore with the potency of ISO (characterized by the negative common logarithm of the half maximal effective ISO concentration) (right panel). Closed symbols indicate samples in the Strong subgroup, whereas open symbols denote samples in the Weak subgroup. Dotted lines show the fitted linear function, while dotted curves represent the 95% confidence interval bands. (Since, there was no statistically significant correlation between parameters indicated in the panels, regression lines and bands are only illustrations).

Both main features of ISO’s effect, the maximal response and the potency (characterized by pEC_50_ being the negative common logarithm of the ISO concentration producing half-maximal response), showed a statistically non-significant negative correlation with the initial contractile force (Pearson r = −0.41, *p* = 0.19; Spearman r = −0.32, *p* = 0.31, respectively) (Figure 6).

### 3.4. Response to BGP-15 and Propranolol

**Figure 7:**
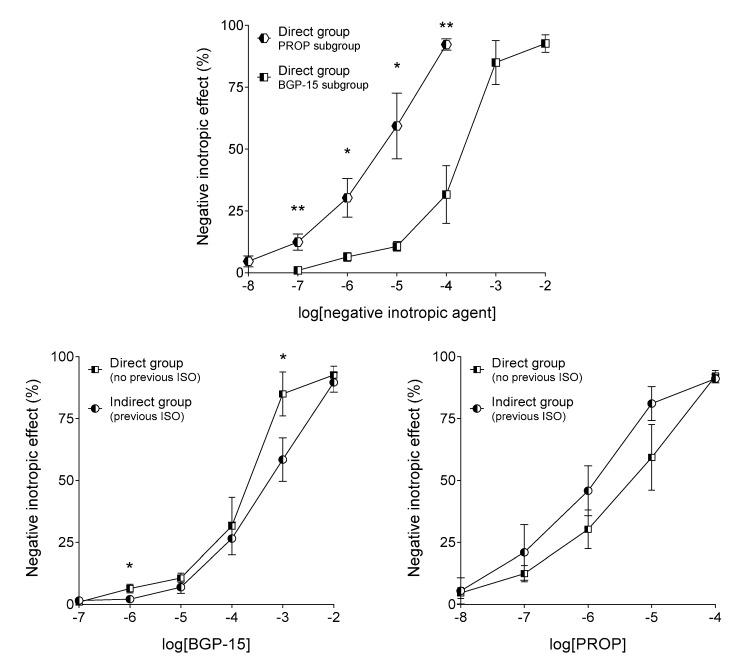
The effect of BGP-15 (upper and left panels) and propranolol (PROP) (upper and right panels) on the human right atrial samples without (Direct group) or with (Indirect group) a previous stimulation with isoproterenol (ISO). The axis *x* denotes the common logarithm of the molar concentration of the chemical, and the axis *y* shows the effect as a percentage decrease of the initial contractile force. The symbols represent the effect averaged within the groups (±SEM). The “Direct group” in the left panel and the right panel means the BGP-15 subgroup and the PROP subgroup, respectively. Of course, the “Indirect group” always refers to the whole Indirect group. Asterisks indicate the significance level of differences between responses of the groups (*: *p* < 0.05; **: *p* < 0.01). SEM: standard error of the mean; BGP-15: O-(3-piperidino-2-hydroxy-1-propyl) nicotinic acid amidoxime hydrochloride.

**Figure 8:**
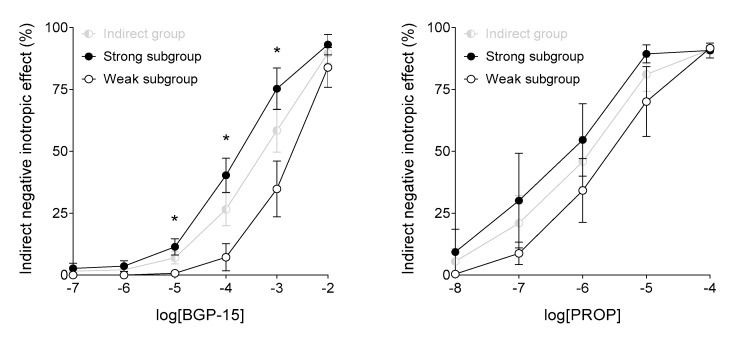
The effect of BGP-15 (left panel) and propranolol (PROP) (right panel) on the human right atrial samples subjected to a previous isoproterenol (ISO) stimulation (Indirect group), dichotomized according to the response to ISO (weak or strong) based on a previously generated ISO concentration-response (E/c) curve. The axis *x* denotes the common logarithm of the molar concentration of the chemical, and the axis *y* indicates the effect as a percentage decrease of the initial contractile force. The symbols represent the effect averaged within the (sub)groups (±SEM). Asterisks show the significance level of differences between responses of the two subgroups of the Indirect group (*: *p* < 0.05). SEM: standard error of the mean; BGP-15: O-(3-piperidino-2-hydroxy-1-propyl) nicotinic acid amidoxime hydrochloride.

In the isolated, paced, human right atrial myocardium, both BGP-15 and propranolol evoked a strong, concentration-dependent, negative inotropic effect (regarding both the direct and indirect ones) (Figure 7 and Figure 8). Beyond the indubitable similarities (most importantly, the practically equal maximal responses), the effect of BGP-15 and propranolol showed some differences.

The main difference between effects of BGP-15 and propranolol is that BGP-15 possessed a considerably smaller potency as compared to propranolol, in terms of both the direct and indirect negative inotropic effects (Figure 7). In addition, BGP-15 seems to have a somewhat stronger direct effect, whereas propranolol appears to exert a stronger indirect action, especially at upper medium concentrations (Figure 7). Furthermore, BGP-15 evoked a substantially greater indirect negative inotropic effect on samples with strong responsiveness to ISO (at medium and upper medium concentrations). In contrast, propranolol did not discriminate significantly between samples with different susceptibility to ISO (although, similarly to BGP-15, it tended to elicit a somewhat greater indirect negative inotropic effect on samples that gave a stronger response to ISO) (Figure 8).

It should be noted here that subgroups of the Direct group are the most suitable for comparing effects of BGP-15 and propranolol, as experimental conditions for these two negative inotropic agents were the same in these subgroups (Figure 2) (for more detail, see below the Section 5).

As shown in Figure 6, ISO was very potent in three atrial samples (see the three data points at the top of the right panel). Since EC_50_ estimation for these three samples was less reliable than for the others, furthermore, since the estimated EC_50_ values were used to stimulate the samples in the Indirect group, the influence of these samples on the BGP-15 and propranolol E/c curve data was tested. Therefore, the evaluation of the BGP-15 and propranolol E/c curves in the Indirect group was repeated with the exclusion of the three “overpotent” samples. This exclusion did not alter the major findings of the evaluation presented so far, leading only two changes worth to mention: differences of BGP-15 E/c curves between the Direct and Indirect groups became statistically non-significant (for the original results, see Figure 7, left panel); and only one propranolol E/c curve remained in the Weak subgroup that hindered the statistical analysis (for the original results, see Figure 8, right panel). Thus, the uncertainty in the estimation of the three EC_50_ values did not meaningfully modify results of the present investigation.

**Figure 9:**
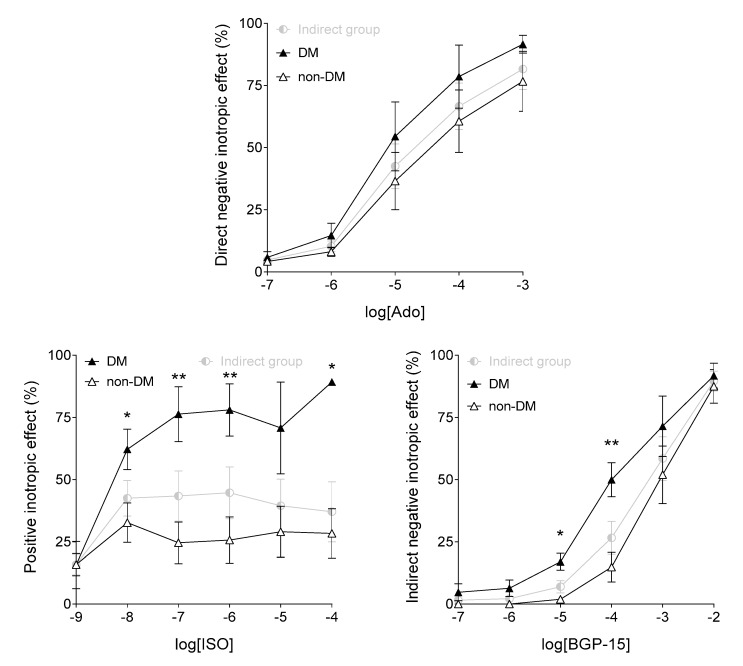
Comparison of effects of adenosine (Ado; upper panel), isoproterenol (ISO; left panel) and BGP-15 (right panel) on the human right atrial samples after dichotomization of data of the Indirect group according to the presence (DM) or absence (non-DM) of known diabetes mellitus (both types). The effect of adenosine is direct, while the effect of BGP-15 is indirect (i.e. occurred in the absence and presence of ISO, respectively). The axis *x* shows the common logarithm of the molar concentration of the agent in question, and the axis *y* indicates the effect as a percentage decrease (Ado, BGP-15) or increase (ISO) of the initial contractile force. The symbols represent the effect values averaged within the data sets (±SEM). Asterisks show the significance level of differences between responses of the diabetic (DM) and non-diabetic (non-DM) data sets of the Indirect group (*: *p* < 0.05; **: *p* < 0.01). SEM: standard error of the mean; BGP-15: O-(3-piperidino-2-hydroxy-1-propyl) nicotinic acid amidoxime hydrochloride.

To explore the influence of diabetes mellitus on the effect of BGP-15, another evaluation was also performed *via* dichotomizing the adenosine, ISO, and BGP-15 E/c data of the Indirect group, according to the presence or absence of diabetes mellitus in the history of patients. The results were astonishingly similar to those obtained by comparing the Weak and Strong subgroups of the Indirect group (cf. Figure 4, right panel with Figure 9, upper panel; Figure 5, right panel with Figure 9, left panel; and Figure 8, left panel with Figure 9, right panel). Thus, ISO and BGP-15 exerted a significantly greater inotropic effect on the diabetic samples than on the non-diabetic ones (Figure 9). The reason for this might be a big overlap of the Strong subgroup with the data set containing the diabetic samples of the Indirect group. Of 12 samples in the Indirect group, 7 gave a strong response to ISO. Of these 7 samples, 4 were derived from diabetic patients. In contrast, of 5 samples producing weak response to ISO, none was obtained from a diabetic patient. Starting from these findings, it was concluded that diabetes mellitus was a major (if not the greatest) factor to increase ISO sensitivity of the atrial samples.

Unfortunately, the limited sample (patient) number did not allow us to evaluate whether type 1 or type 2 diabetes mellitus (or both ones) is (are) responsible for the above-mentioned phenomenon. Nevertheless, occurrence of the different types of diabetes mellitus in our database shows the dominance of the type 2: 7/9/22 (type 2 diabetes mellitus/all diabetes mellitus/all patients, for the pooled Direct and Indirect groups), and 3/4/12 (the same solely for the Indirect group).

### 3.5. Associations between Patient Data and Sample Features

During the recruitment period, 30 patients were included (7 females and 23 males) ranging from 29 to 78 years (59.5 ± 12.4). From the 30 samples obtained, 26 proved to be technically sound. Of them, 10 samples, derived from patients (3 females and 7 males) ranging from 40 to 70 years (58.7 ± 9), were randomized into the Direct group, while 16 samples, obtained from patients (2 females and 14 males) ranging from 29 to 76 years (57.9 ± 13.5), were sorted into the set receiving ISO. Of this latter set, 12 patients (1 female and 11 males) from 29 to 76 years (58.7 ± 15.4), who provided atrial samples giving “unextreme” response to ISO (i.e., the effect of ISO was neither negative nor extremely strong), formed the Indirect group.

The relationship of the occurrence of certain conditions (yes or no) with the naïve contractility (small or large), and with the response to ISO (weak or strong) were assessed (Table 1).

All patients suffered from several diseases and took numerous drugs. From these factors, NO donors and trimetazidine appeared to beneficially influence the contractile force of the atrial samples. Furthermore, diabetes mellitus and hypertension seemed to increase, while proton-pump inhibitors tended to decrease the response to ISO. From these associations, none proved to be statistically significant (Table 1).

## 4. Discussion

The increasing prevalence of CVDs can be largely attributed to behavioral risk factors such as smoking, sedentary lifestyle, obesity, and excess alcohol consumption [1,23,24,25]. Although in early stages of CVDs, lifestyle changes may be sufficient, in advanced stages, pharmacotherapy is inevitable. It should be emphasized, however, that all the widely available therapeutic options have their own limitations in terms of mild to severe adverse effects and drug interactions [26,27]. Therefore, in this study, we focused on BGP-15, a drug candidate, which possesses many cardioprotective effects [19,20,21,28] and, based on a phase II investigation, proved to be safe for human use [29].

BGP-15 exerts many kinds of effects. However, despite the endeavor of several studies, cardiac effects of BGP-15 are still not completely explored. It has been established that BGP-15 exerts several protective effects by inducing the expression of HSP70 or HSP72 [13,14,15,16,17,18,30,31], but these studies dealt mostly with metabolic syndrome. In addition, others have found that BGP-15 preserves muscle function by increasing the level of HSP72 in skeletal muscles [32,33]. On the other hand, Sapra et al. did not find elevated HSP70 levels in the myocardium after BGP-15 treatment [19], suggesting that cardiac effects of this compound are mediated independently of HSP70. Moreover, the poly(ADP-ribose) polymerase inhibitor property of BGP-15 was also reported to protect the heart against ischemia-reperfusion injuries by reducing reactive oxygen species [20,21].

Furthermore, Sapra et al. have found that phosphorylation of insulin-like growth factor 1 receptor (IGF1R) is increased after BGP-15 treatment. IGF1R, a member of the insulin receptor family, participates in the mediation of protective effects against numerous CVDs. It should be noted that phosphorylation of IGF1R is suppressed in atrial fibrillation and heart failure [19]. In addition, a relationship was found between the phosphorylation of IGF1R and the level of monosialodihexosylganglioside (GM3), a glycosphingolipid related to insulin receptors. Increased level of GM3 attenuates the insulin receptor signal transduction [34,35], and it may also contribute to some atrial maladies [19].

In connection with all these mechanisms of action, BGP-15 also acts by remodeling the lipid rafts, facilitating the actions of membrane-localized receptor proteins via enhancing membrane fluidity [31,36]. Returning to Sapra et al., membrane-localized IGF1R [37] and increased GM3 levels may reduce membrane fluidity [19].

In addition, our research team has previously found that BGP-15 improves diastolic function in Goto-Kakizaki rats by increasing the phospholamban phosphorylation, which leads to enhanced SERCA2A (sarcoplasmic/endoplasmic reticulum calcium ATPase) pump activity [38].

To the best of our knowledge, the present study is the first dealing with the inotropic action of BGP-15, and compared it to that of propranolol, the prototypical non-cardioselective β-adrenergic antagonist with additional membrane stabilizing activity (when used at high concentrations) [39]. The relevance of this comparison stems from the fact that BGP-15 and propranolol have a significant structural similarity, so BGP-15 may exert β-blocker activity. In our model using isolated, paced, human atrial trabeculae carneae, contractility can be reliably examined, without the disturbing influence of the chronotropic effect. Although contractility of the left ventricle is one of the most important parameters of the cardiac function, investigation of the atrial myocardium is also useful. Atrial booster pump function has a significant role in diastolic ventricular filling, especially in the elderly [40,41]. Moreover, inappropriate mechanical activity of atria, leading to inadequate atrial emptying, may even promote blood clotting, as has been found in atrial fibrillation that increases the risk of systemic thromboembolism and stroke five-fold [42].

In the present study, we observed that BGP-15, at low concentrations (≤10 μmol/L), exerted a negligible negative inotropic effect (including its direct and indirect types), while it elicited robust negative inotropy at higher concentrations (≥1 mmol/L). For comparison, at 10 μmol/L, propranolol decreased the contractile force to less than half of the initial value (Figure 7). Thus, although their maximal effect was similar, the potency (−logEC_50_ = pEC_50_) of BGP-15 was considerably smaller than that of propranolol (regarding the ability to decrease the human atrial contractile force, in terms of both types of negative inotropy) (Figure 7).

In adult patients, BGP-15 was found to exert an insulin-sensitizing effect at 2–3 mg/kg/day doses [9,10,11,12] that may produce BGP-15 concentrations in micromolar order of magnitude in the body. Taking this observation together with that BGP-15 is ineffective as a negative inotropic agent until 10 μmol/L (Figure 7), it can be concluded that beneficial metabolic effects of BGP-15 will not be accompanied by a significant decrease in contractility (regarding atria, and maybe ventricles as well).

In the intermediate range (between 10 μmol/L and 1 mmol/L), the indirect negative inotropic effect of BGP-15 significantly depended on the atrial responsiveness to ISO: only samples exhibiting strong positive inotropic response to ISO produced a considerable negative inotropic response to BGP-15 (Figure 8). In contrast, propranolol’s action was less influenced by the β-adrenergic responsiveness of the atrial samples, although samples with a greater susceptibility to ISO gave a (statistically non-significantly) stronger response to propranolol (Figure 8). This appears to be a beneficial property in favor of BGP-15, because it may reduce the cardiac function stimulated by an increased sympathetic tone to a greater extent than the resting activity of the heart. This phenomenon shows some abstract similarities to the negative chronotropic effect of local anesthetics, i.e., they can effectively slow the heart in tachycardia but not in bradycardia (exerting a so-called use-dependent effect).

To consider the above-mentioned observation, it should be pointed out that ISO, a synthetic β-adrenergic receptor agonist, is a widely used agent to induce experimental cardiac hypertrophy, heart failure, or even myocardial infarction [43,44,45,46]. Consistently, ISO increases the level of free radicals leading to oxidative stress in the myocardium [3]. Moreover, ISO can mimic acute and chronic stress for human peripheral blood mononuclear cells [47].

In addition, we investigated the relationship between functional properties of the atrial samples (contractile force, response to ISO) and data of the patients (gender, underlying health conditions, medications used). It should be noted that the patients had multiple diseases and took several drugs, rendering this investigation difficult. Nevertheless, the strong response to ISO seems to be associated with hypertension and diabetes mellitus (Table 1). Regarding this latter association (i.e., the enhanced ISO sensitivity with the diabetic condition), it should be noted that most of the strong ISO responder samples were obtained from diabetic patients (predominantly suffering from type 2 diabetes mellitus). Thus, it is reasonable to conclude that the response to ISO has an inverse relationship with the “fitness” of the atrial myocardium. This conclusion is further supported by the fact that all pathological conditions, investigated in the current study, tended to increase the response to ISO (Table 1).

Consistently, in the present investigation, the susceptibility to ISO seems to be a better measure of functionality of the atrial myocardium than the naïve contractility. This can be due to that, in our model, contractile force is determined by additional factors unrelated to the viability of the myocardium (e.g., size and shape of the trabecula carnea). These factors may weaken the strength of the statistical relationship between the myocardial functionality and the resting contractile force.

Conversely, when contrasted the behavior of atrial samples obtained from diabetic vs. non-diabetic patients, the results showed convincing similarity to the outcome of the comparison of samples giving strong vs. weak response to ISO (cf. Figure 4, right panel with Figure 9, upper panel; Figure 5, right panel with Figure 9, left panel; and Figure 8, left panel with Figure 9, right panel). Thus, BGP-15 exerted a significantly stronger indirect negative inotropic effect on the diabetic atrial samples than on the non-diabetic ones (Figure 9, right panel). This finding corresponds with the results of others, who found a bidirectional relationship between insulin resistance and enhanced β-adrenergic stimulation in the heart [48,49]. This relationship may stem from a mutual counter-regulation between signaling pathways of insulin and β-adrenergic agonists. This mechanism may underly the ability of β-blockers to ameliorate insulin resistance in the heart [48,49]. Applying cardioselective (or a fortiori cardioselective plus vasodilatory) β-blockers, this insulin-sensitizing effect can be even therapeutically exploited [50].

In summary, BGP-15 is a negative inotropic agent with a considerably smaller potency in comparison with propranolol, the parent molecule. Another difference between effects of BGP-15 and propranolol is that BGP-15, in contrast to propranolol, decreased the contractility of atrial samples showing strong response to ISO to a significantly greater extent than it did regarding samples with weak responsiveness to ISO. Overall, our present functional results suggest that the mechanisms of the negative inotropic action for BGP-15 and propranolol may in part differ from each other. In addition, diabetes mellitus seems to be associated with an increase in the responsiveness to ISO, so enhanced ISO sensitivity may be a disadvantageous condition for the atrial myocardium. Moreover, the diabetic state may be one of the most important factors to influence functionality (and to deteriorate viability) of the myocardium (in the atrium, and possibly in the ventricle as well). It has also been found that contractility of samples stimulated with ISO is significantly more sensitive to BGP-15, if obtained from diabetic patients (in contrast to those derived from non-diabetic patients).

## 5. Study limitations

The major limitation of the present work stem from the features of the study design (Figure 2). The major aim of the present study was to collect reliable data as much as possible about the inotropic effect of BGP-15 on the human atrial myocardium. Therefore, in the Indirect group, the E/c curves constructed with BGP-15 always preceded the ones generated with propranolol that finally resulted in relatively few data on propranolol. (In the Indirect group, 12 BGP-15 E/c curves and 7 propranolol E/c curves were generated. The smaller number of propranolol E/c curves came from the exhaustion of some of the samples by the end of this protocol).

A further consequence of the study design is that while propranolol did not affect the data on BGP-15 at all, some influence of BGP-15 on data about propranolol in the Indirect group cannot be excluded. Thus, results with propranolol in the Indirect group should be treated with some caution, e.g., when comparing them with data on BGP-15 of the Indirect group, or with data on propranolol in the Direct group.

Another limitation may concern the estimation of ISO EC_50_ values for some atrial samples, i.e., for those, response of which to ISO proved to be very potent. Namely, EC_50_ estimation for three samples (see the three data points at the top of the right panel of Figure 6) was less reliable than for the others. The impact of this stems from the fact that ISO EC_50_ values were used to stimulate the samples in the Indirect group, thus any inaccuracy in the ISO EC_50_ values might influence the subsequent BGP-15 and propranolol E/c curves. However, according to the re-evaluation of data in the Indirect group with the exclusion of the affected three samples, this uncertainty appears not to meaningfully modify results and conclusions of the present investigation.

It would had been interesting to compare the effects of propranolol on the diabetic and non-diabetic samples. Unfortunately, it was hindered by the lack of enough diabetic samples reflecting the effect of propranolol. This happened even though that, in the other evaluational arrangement (strong vs. weak ISO responder samples), there was a sufficient quantity of strong responder samples to obtain technically sound results on propranolol. This was due to that, in the Indirect group, the reduction in the sample number before the construction of the propranolol E/c curve affected predominantly the diabetic samples. So, among samples providing strong response to ISO, the non-diabetic ones proved to be more viable and were concentrated until the beginning of the propranolol E/c curve.

Due to the limited sample number, the issue, whether type 1 or type 2 diabetes mellitus (or both ones) is (are) responsible for this phenomenon, could not be properly addressed herein. Nevertheless, in our database, occurrence of type 2 diabetes mellitus exceeded that of type 1, so the major role of type 2 diabetes mellitus can be assumed. Furthermore, the small sample number in the Direct group hindered the technically sound comparison between the diabetic and the non-diabetic samples.

## 6. Conclusions

The major finding of the present study is that BGP-15, a derivative of the non-selective β-blocker (and, to a smaller extent, membrane-stabilizing) propranolol, exerted both direct and indirect negative inotropic effects on the *ex vivo* human right atrial myocardium. However, the negative inotropy elicited by BGP-15 can be characterized with a considerably smaller potency than that exerted by propranolol, although they possess practically the same maximal value. Thus, it is reasonable to conclude that insulin-sensitizing effects of BGP-15 may develop without a significant negative inotropy as a side effect. Furthermore, the fact that BGP-15 elicited a greater negative inotropic effect on samples showing a strong response to ISO may also be beneficial because enhanced beta-adrenergic sensitivity can accompany type 2 diabetes mellitus, in which it may contribute to the maintenance of insulin resistance. It is consistent with another finding of the present study, i.e., BGP-15 had a greater indirect negative inotropic effect on samples obtained from diabetic patients. In addition, pharmacological properties of BGP-15 might be tested in the treatment of heart failure, a condition also associated with chronic sympathetic overactivity leading to detrimental cardiac remodeling. These latter conclusions, of course, are based on the assumption that a greater effect on contractility is associated with greater beneficial effects regarding BGP-15.

## Figures and Tables

**Table 1:** Relationship of gender, diseases, and medications of patients with the magnitude of the contractile force of atrial samples (measured at the end of the wash-out period after the third adenosine concentration-response curve), and with the magnitude of the response to isoproterenol (ISO).

	Contractile Force (Direct + Indirect; *n* = 22)		*p*	Response to ISO (Indirect; *n* = 12)		*p*
	Small (*n* = 13) %	Large (*n* = 9) %		Weak (*n* = 5) %	Strong (*n* = 7) %	
**Gender** (female)	15.4	22.2	>0.99	0	14.3	>0.99
**Diseases**						
DM	46.2	33.3	0.67	0	57.1	0.08
Hyperlipidemia	46.2	44.4	>0.99	40	57.1	>0.99
Ischemia	84.6	77.8	>0.99	40	85.7	0.22
Hypertension	69.2	77.8	>0.99	20	85.7	0.07
Heart failure	46.2	33.3	0.67	20	71.4	0.24
VHD	46.2	33.3	0.67	20	57.1	0.29
**Drugs**						
Antiplatelets	76.9	66.7	0.66	60	42.9	>0.99
Anticoagulants	38.5	33.3	>0.99	42.9	14.3	0.56
β-blockers	84.6	100	0.49	100	71.4	0.47
ACEI	61.5	88.9	0.33	60	71.4	>0.99
Ca^2+^ channel b.	15.4	22.2	>0.99	20	14.3	>0.99
NO donors	15.4	44.4	0.18	0	14.3	>0.99
Diuretics	46.2	66.7	0.41	40	57.1	>0.99
Trimetazidine	15.4	44.4	0.18	0	28.6	0.47
Statins	76.9	66.7	0.66	40	57.1	>0.99
Insulin	15.4	11.1	>0.99	0	28.6	0.47
Oral antidiab.	30.8	22.2	>0.99	28.6	0	0.47
PPI	69.2	44.4	0.38	100	57.1	0.2
Potassium	23.1	22.2	>0.99	20	28.6	>0.99
Benzodiazep.	15.4	22.2	>0.99	20	14.3	>0.99
Allopurinol	7.7	22.2	0.54	0	14.3	>0.99

The percentage occurrence of patients with a given condition regarding the contractile force and the response to ISO, with the related level of statistical significance, are indicated. Small: small contractile force; Large: large contractile force; Weak: weak positive inotropic response to ISO; Strong: strong positive inotropic response to ISO; Direct: Direct group; Indirect: Indirect group; DM: diabetes mellitus (both types); Ischemia: chronic ischemic heart disease, angina pectoris, myocardial infarction (with or without ST-elevation), ischemic cardiomyopathy; Heart failure: cardiac decompensation, congestive heart failure, dilated cardiomyopathy; VHD: valvular heart disease; ACEI: angiotensin-converting enzyme inhibitors; Ca^2+^ channel b.: blockers of the L-type calcium channel; NO donors: compounds that provide nitric oxide in the body (e.g., nitrites and nitrates); Oral antidiab.: orally active antidiabetic drugs; PPI: proton-pump inhibitors; Benzodiazep.: benzodiazepines.
